# Genetic diversity of the pampas deer (*Ozotoceros bezoarticus*) population in the Brazilian Pantanal assessed by combining fresh fecal DNA analysis and a set of heterologous microsatellite loci

**DOI:** 10.1590/1678-4685-GMB-2016-0323

**Published:** 2017-10-02

**Authors:** Aline Meira Bonfim Mantellatto, Renato Caparroz, Maurício Durante Christofoletti, Ubiratan Piovezan, José Maurício Barbanti Duarte

**Affiliations:** 1 Núcleo de Pesquisa e Conservação de Cervídeos, Departamento de Zootecnia, Universidade Estadual Paulista “Júlio de Mesquita Filho” (UNESP), Jaboticabal, SP, Brazil; 2 Departamento de Genética e Morfologia, Instituto de Ciências Biológicas, Universidade de Brasília, Brasília, DF, Brazil; 3 Centro de Pesquisas Agropecuárias do Pantanal (CPAP), Embrapa, Corumbá, MS, Brazil

**Keywords:** Conservation, fecal DNA, microsatellites, non-invasive methods, pampas deer

## Abstract

The pampas deer (*Ozotoceros bezoarticus*) is close to being classified as ‘globally threatened’, with the largest population occurring in the Brazilian Pantanal. Since capture is stressful to these animals, non-invasive sampling methods such as the use of feces can provide reliable sources of DNA. The aim of this study was to use fecal samples to evaluate the genetic variability of the Brazilian Pantanal population of pampas deer. Six heterologous microsatellite markers were used to screen 142 stool specimens. Seventy-four deer were identified, of which 50 adults were used to determine the genetic characteristics of the population. The Pantanal population showed high genetic diversity (mean number of alleles per locus = 11.5, expected heterozygosity = 0.75). This is the first investigation to characterize a South American deer species using fecal DNA and demonstrates the usefulness and efficiency of this approach, as well as the feasibility of obtaining information that could not have been easily obtained by traditional DNA sampling. Our findings suggest that management strategies for this species may be much more effective if applied now when the population still shows high genetic variability.

## Introduction

Population genetic studies of wild populations of species of the family Cervidae have become feasible with the advent of fecal DNA analysis (Lounsberry *et al.*, 2015; Yamashiro *et al.*, 2015), particularly since these species have elusive habits and are usually listed as locally, nationally and/or globally endangered species. The capture of wild animals is potentially very stressful and can cause injuries (Greenwood, 1996; Duarte *et al.*, 2010) with capture-related myopathy being frequently reported for species of this family (Catão-Dias and Camargo, 2010; Duarte, 2008). The fecal DNA technique allows researchers to economize financial resources and time, since conventional capture expeditions are extremely expensive and provide insufficient samples for population studies (Duarte, 2006).

The pampas deer (*Ozotocerus bezoarticus*) is a medium sized (20-40 kg) Neotropical cervid with marked variation in body size among individuals and populations (González *et al.*, 2010). The original distribution of this species included the Pampas region and practically all of the Brazilian Cerrado, from 5º to 41º south latitude, from the foothills of the Andean system to the Atlantic coast (Duarte, 2006; González *et al.*, 2010). Although the pampas deer has historically had a wide geographical distribution, its habitat has been intensely reduced and fragmented by agriculture and urbanization, making this species the most endangered neotropical cervid (González, 1998).

To date, five subspecies of *O. bezoarticus* have been recognized based on morphological, genetic and geographical differences (Cabrera, 1943; González *et al.*, 2002). The total population of *O. bezoarticus leucogaster* is estimated at 60,000 individuals (Mourão *et al.*, 2000) and occurs in an area of 151,000 km2 (58,300 mi2) covering southwestern Brazil, Bolivia, Paraguay and northern Argentina. This subspecies is classified as vulnerable to extinction because of its likely future decline (estimated at 30% in the next 15 years), attributable primarily to the introduction of pathogens via domestic ungulates (Duarte *et al.*, 2001; Araújo Júnior *et al.*, 2010) and to the tendency of substituting natural pastures (Duarte *et al.*, 2012). According to the IUCN Red List, *O. bezoarticus* is considered near threatened; however, the populations in Argentina, Bolivia, Paraguay and Uruguay are considered endangered (González *et al.*, 2016). Given the reduction and modification of their habitats, populations of pampas deer are becoming smaller and more isolated, with both of these phenomena increasing the species risk of experiencing reduction of genetic variability (González *et al.*, 1998). Genetic variability can be rapidly lost in a population that remains small for many generations, raising the possibility of extinction of these populations (Frankhan *et al.*, 2004).

To date, few studies have examined the genetic diversity of *O. bezoarticus* populations. González *et al.* (1998) examined sequences of the mitochondrial DNA control region in 54 individuals from six locations within the current distribution of pampas deer and verified that the surviving populations are small, isolated and genetically differentiated, except for two populations in Argentina. Furthermore, these same authors pointed out that the genetic variability of the control region observed in this species was one of the highest among mammals, suggesting that historically the population size of this species was much larger (estimated in millions of individuals) than it is today (estimated at < 60,000, Mourão *et al.*, 2000). In contrast, Rodrigues *et al.* (2007) studied the same two Brazilian populations studied by González *et al.* (1998) and found no genetic difference between them as assessed using nuclear molecular markers. These authors attributed the divergent results to genetic differences between the markers (mitochondrial DNA vs RAPD) used and male-biased dispersal. They also concluded that more studies using other nuclear molecular markers (such as microsatellites) are needed before genetic data can be used to guide conservation measures for this species. Cossé *et al.* (2007) and Cossé (2010, PhD thesis, Universidade de la Republica, Uruguay) described the only work that has used microsatellite molecular markers to genetically characterize the six populations of pampas deer in Uruguay, Argentina and Brazil, and the results corroborated the high genetic diversity of the species. Although Cossé (2010, PhD thesis, Universidade de la Republica, Uruguay) genetically characterized samples from 100 pampas deer, only seven of these were from individuals belonging to the Brazilian Pantanal population, which currently has the highest concentration of this species both in absolute numbers (Mourão *et al.*, 2000) and in population density (Tomas *et al.*, 2001).

In this study, we investigated the genetic variability of a population of *O. bezoarticus leucogaster* from the Brazilian Pantanal region by combining a non-invasive technique based on fresh fecal DNA analysis and the use of nuclear microsatellite markers. To our knowledge, this is the first study to associate these methodologies in an investigation of native ungulates in Brazil. Our main objectives were to evaluate the effectiveness of using fresh fecal samples and analyze a larger number of individuals in order to confirm that the studied population had high genetic diversity, as previously described.

## Materials and Methods

### Sample collection and DNA extraction

**Figure 1:**
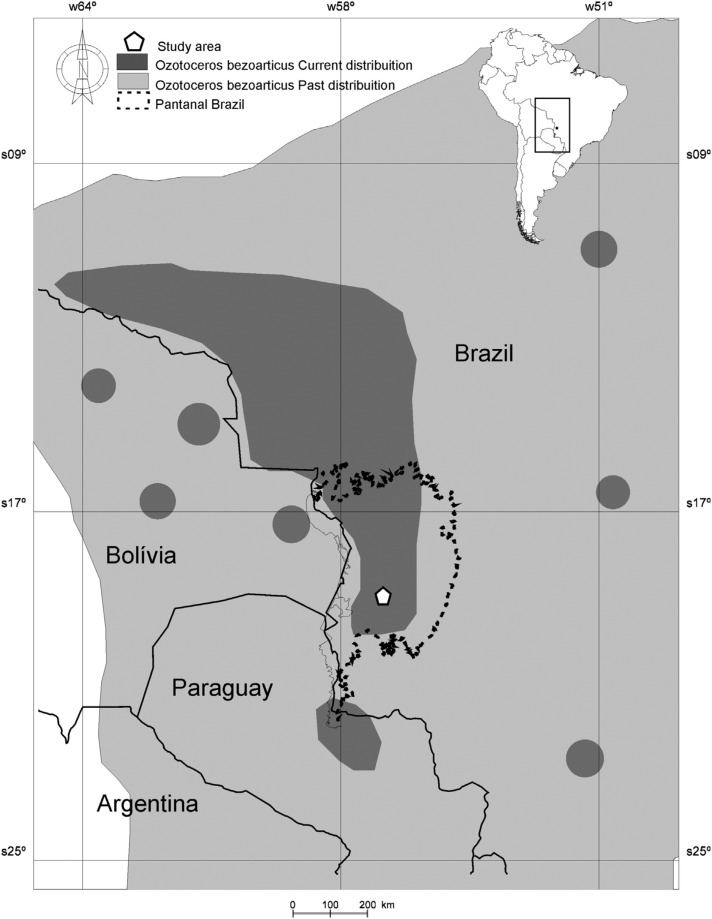
Map showing the past and current distribution of the pampas deer and the predominant distribution in Brazil. The location of the population studied in this work is highlighted.

The stool sample collection area included the Nhumirim (experimental field of Embrapa Pantanal) and Alegria farms, located in the central region of the Brazilian Pantanal, known as Nhecolândia (18º59′15″ S; 56º37′03″ N) (Figure 1). Deer were observed until they defecated in order to collect fresh fecal samples. All of the individuals sampled were classified in one of the following categories according to sex and age: adult male, young male, adult female, young female and undetermined. One hundred and forty-two fecal samples were collected. These samples were identified and stored in plastic bags and refrigerated (4 °C) in a cooler with ice packs until they arrived at base camp, where they were frozen (-20 °C) and stored until processing in the laboratory. Total genomic DNA was extracted from the stool samples using QIAamp^®^ DNA Stool mini kits (Qiagen), according to the manufacturer's recommendations.

### Heterologous primers

The transferability of 11 heterologous primer pairs (Table 1) was evaluated in 13 blood samples from adult female *O. b. leucogaster* captured in the study area. The markers were selected because they showed cross-amplification with other Neotropical deer species, while seven of them (RT01, RT06, RT07, RT09, RT13, RT30 and CA71) had proven transferability to five species of *Mazama* (Mantellatto *et al.*, 2010), three (NVHRT01, NVHRT03 and NVHRT16) to the marsh deer (Leite *et al.*, 2007) and one (BM757) to *O. bezoarticus* (Cossé *et al.*, 2007).

**Table 1:** Summary of the 11 microsatellite loci included in the characterization of 13 female pampas deer (*Ozotoceros bezoarticus leucogaster*) from the Brazilian Pantanal using heterologous primers.

Locus	Fluorescence	Repetition	T_A_ (°C)	N_A_	Range (bp)
RT01^1^	Fam	(GT)_22_	55.5	5	220-230
RT06^1^	——	(GT)_23_	55.5	3	120-130
RT07^1^	——	(GT)_18_	55.5	4	190-200
RT09^1^	Fam	(GT)_21_	55.5	5	120-130
RT13^1^	——	(GT)_13_	55.5	3	290-296
RT30^1^	Hex	(GT)_21_	55.5	5	200-220
NVHRT01^2^	Ned	(GT)_7_GC(GT)_12_	50.0	5	164-200
NVHRT03^2^	Ned	(CT)_7_TA(CA)_12_	50.0	5	112-126
NVHRT16^2^	Hex	(CA)_5_TA(CA)_5_(TG)_2_CG(CA)_19_	50.0	7	152-192
CA71^3^	Hex	(CT)_12_	50.0	3	300-310
BM757^4^	Fam	(GT)_17_	58.5	7	195-227

N_A_ – number of alleles, T_A_ – annealing temperature in degrees Celsius, —— no fluorescence, *i.e.*, locus was not used in genotyping. References:

1 Wilson *et al.* (1997),

2 Roed and Midthjell (1998),

3 Gaur *et al.* (2003),

4 Bishop *et al.* (1994).

The PCR reactions were standardized to a final volume of 15 μL, containing 1X of *Taq* buffer (10 mM Tris-HCl pH 8.4, 50 mM KCl, 2 mM MgCl_2_), 120 μM of dNTPs, 0.4 U of *Taq* polymerase (Invitrogen), 45 ng of genomic DNA and 0.08 mM of each primer. The same temperature cycles were used for all primers, with variation only in the annealing temperature, as follows: 94 °C for 5 min, 94 °C for 1 min, 50-59 °C for 1 min (depending on the primer pair shown in Table 1), 72 °C for 1 min and 72 °C for 10 min. The PCR products were applied to a denaturing polyacrylamide gel and stained with 10% silver nitrate to evaluate the polymorphism at each locus. After confirming transferability, the loci for individualizing fecal samples collected in the field were selected based on polymorphism (more than three alleles) and the quality and sharpness of the allele bands on the gels.

### Genotyping of fecal samples

To individualize the 142 fecal samples collected, fluorescent labeling of the PCR products was done by adding fluorescence (HEX, NED or FAM) to one of each primer pair. The reaction had a final volume of 11 μL, containing 0.5 μM of each primer, 1X buffer, 0.3 μL of MgCl_2_, 0.6 μL of BSA, 1.0 μL of dNTPs, 1.0 unit of *Taq* polymerase (Platinum^®^ Invitrogen) and 2.6 μL of ultrapure water. The remainder of the 11 μL reaction volume was completed with DNA sample. The amplicons were then run on ABI 3100 and ABI 3130 automatic sequencers and assigned genotypes using the program GeneMarker v.2.4.2 (SoftGenetics LLC).

To overcome problems such as allelic dropout and false alleles (Taberlet *et al.*, 1996; 1999), the reactions and genotyping were done in duplicate for all samples. When the result obtained was inconsistent with its respective replicate, triplicate and quadruplicate tests of the same samples were run to minimize these genotyping errors. This was done for about 21% of the total samples, 50% of these belonging to the locus BM757. The deer were individualized by comparing the genotypes obtained from each fecal sample and considering only those with positive results for at least three microsatellite loci. Since some studies have shown that the success in amplification varies according to locus size (*e.g.* Oliveira and Duarte, 2013), we also investigated the correlation between these variables by calculating Pearson's correlation coefficient.

### Genetic characterization of the population

For the next analysis, we considered only the genotypes of adult deer individualized in the previous step, thereby avoiding overlapping generations. The Hardy-Weinberg equilibrium (HWE), expected (*H*_*E*_) and observed heterozygosity (*H*_*O*_), and analyses of linkage disequilibrium were calculated using the program Genepop 1.2 (Raymond and Rousset, 1995), with the following parameters for the Markov chain Monte Carlo (MCMC): dememorization 10,000, batches 1,000, and iterations per batch 10,000. The Bonferroni correction (Rice, 1989) was applied to the nominal value of α = 0.05 to adjust the levels of significance in the analysis. The presence of errors in the genotyping was assessed using the Micro-Cheker program (Van Oosterhout *et al.*, 2004). The probability of genetic identity (*P*_*ID*_), which corresponds to the probability of two random individuals exhibiting the same genotype (Chakravarati and Li, 1983), and the probability of paternity exclusion (*Q*), which corresponds to the force with which a locus excludes an individual from being the mother of an offspring (Weir 1996), were estimated using the program Identity 1.0 (Wagner and Sefc, 1999).

## Results

### Cross-amplification

All pampas deer loci tested showed successful cross-amplification and were polymorphic since more than two alleles per locus were evident in blood samples from the 13 females studied (Table 1). Six loci (CA71, BM757, NVHRT16, NVHRT03, RT01 and RT09) attained our quality criteria (see *Heterologous primers* section) and were used for fecal sample individualization.

### Individualization of fecal samples

At least one microsatellite locus was amplified from the 142 fecal samples analyzed, but the six selected loci were amplified in only 40.8% of the samples. Considering our selection criteria (sample genotyping for more than three loci), only 131 samples were used in the sample individualization step. The latter analysis allowed us to identify 74 deer (50 adults, 19 young and 5 undetermined) during the study period.

The six loci used showed an amplified microsatellite fragment size of 112-310 bp (Table 1). There was no correlation between successful amplification and microsatellite locus size (R^2^ = 0.0082 and *p* = 0.86). The smallest amplified fragment (locus NVHRT03, ranging from 112-126 bp) showed 76.8% successful amplification in the 142 samples tested, while the largest fragment (locus CA71, ranging from 300-310 bp) was successfully amplified in 69% of samples. The locus with the greatest success in amplification (93.7%) was RT01 (220-230 bp).

### Genetic characterization of the population

Based on the analysis of 50 samples from adult deer (27 males and 23 females, see Table S1) in which there were no overlapping generations, only locus CA71 showed Hardy-Weinberg disequilibrium (Table 2), probably because of the presence of null alleles. For the remaining loci, no null, stutter or dropout alleles were detected. All the loci showed linkage disequilibrium. The mean number of alleles was 11.5 and the mean expected heterozygosity (H_E_) was 0.75 (Table 2). The mean probability of identity (P_ID_) obtained for the six loci was 1.74 × 10^−8^, while the mean probability of paternity exclusion (*Q*) was > 99%.

**Table 2:** Characterization of six microsatellite loci transferred to *Ozotoceros bezoarticus leucogaster* based on the genotyping of 50 individuals in a population from the Brazilian Pantanal.

Locus	N_A_	HWE	*H*_*E*_	*H*_*O*_	*Q*	*P*_*ID*_
RT01	13	0.65	0.86	0.84	0.74	0.03
RT09	6	0.57	0.70	0.69	0.45	0.14
BM757	17	0.49	0.90	0.92	0.80	0.02
NVHRT03	11	0.03	0.86	0.76	0.73	0.03
NVHRT16	17	0.62	0.90	0.90	0.80	0.02
CA71	5	0.00	0.29	0.24	0.16	0.51
Mean	11.5	0.39	0.75	0.72	0.99	1.74 × 10^−8^

HWE – Probability values obtained by the H-W equilibrium test, *H*_*E*_ – expected heterozygosity, *H*_*O*_ – observed heterozygosity, N_A_ – number of alleles, *Q* – probability of paternity exclusion and *P*_*ID*_ – probability of genetic identity.

## Discussion

By combining a non-invasive method (fecal DNA analysis) with the use of microsatellite markers we conducted the first population genetic study on a large sample of pampas deer in the Brazilian Pantanal, in Mato Grosso do Sul, home to the largest population of this species.

The six loci studied were amplified in at least 69% of the samples, more than required for deer individualization analysis, for which at least three loci per sample were amplified, as recommended by Miotto *et al.* (2011). The total probability of genetic identity (*P*_*ID*_) was close to 99%, indicating that the six loci were more than adequate to individualize the fecal samples and that the individualization of 74 deer based on these loci was reliable. Furthermore, the low value for the probability of paternity exclusion for all the loci showed that they formed a set of appropriate codominant markers for paternity/maternity and genetic studies in pampas deer populations.

The amplification of at least one microsatellite locus in all the samples and 5-6 loci in ~70% of the samples analyzed indicated a high rate of success for fecal DNA. These results indicate that the use of fresh fecal samples can enhance the success of studies using fecal DNA samples (Oliveira and Duarte, 2013). In contrast, based on samples collected in northern Chile during the summer, Espinosa *et al.* (2015) concluded that fresh feces (based on the presence of mucus or deer observed defecating) and non-fresh feces (no mucus, deer not observed defecating) were equally efficient for successful DNA amplification in ungulate species.

The data obtained here revealed high genetic diversity in the pampas deer population of the Brazilian Pantanal (Table 2). As expected, the mean number of alleles per locus was higher with the increase in sample size. The mean number of alleles per locus for the Pantanal population (n = 50) was 11.5, but was 5.8 based on the genotypes of five individuals from the same population (Cossé 2010, PhD thesis, Universidade de la Republica, Uruguay) (for genotypes, see page 190). On the other hand, the expected mean heterozygosity (H_E_ = 0.75) for the Pantanal population (n = 50) was slightly lower than that reported (Cossé M, 2010, PhD thesis, Universidade de la Republica, Uruguay) (H_E_ = 0.87, n = 5), suggesting that the genetic variability of the Pantanal population is slightly lower than previously described for the same type of genetic marker. The high genetic diversity observed in this population of pampas deer was also observed in a study of dominant nuclear markers of random amplified polymorphic DNA (RAPD) (Rodrigues *et al.*, 2007). Studies using mitochondrial markers from the D-loop control region (González *et al.*, 1998; Braga *et al.*, 2005) and from cytochrome b (Braga *et al.*, 2005) also confirmed the high nucleotide diversity of the species. Consequently, González *et al.* (1998) suggested that the control region in pampas deer is one of the most polymorphic among mammals and that the large number of haplotypes indicates this was once a much more abundant species of deer.

The high genetic diversity reported here strongly supports the relevance of this *O. bezoarticus* population. The high quality habitats of this population are changing in response to political and economic policies that encourage the installation of major development projects, the conversion of natural landscapes to allow the introduction of exotic grasses and a variety of other practices. One example of the latter is the deforestation of areas of mountain ranges, with a phytophysiognomy characterized by sandy ridges under Cerrado vegetation, that are one or two meters above the level of the fields, so they generally do not flood (Junk and Silva, 1999). Another is the systematic burning of the Caronal, a phytophysiognomy characterized by savanna vegetation predominantly covered by wild lemongrass (*Elyonurus muticus*), to establish and improve grazing pastures (Cardoso *et al.*, 2000a,b; 2003). These examples raise concern regarding the conservation of the wild populations that inhabit these biomes (Cunha and Junk, 2009).

Based on the set of nuclear microsatellite markers evaluated here, further research should be done to compare the genetic diversity of different populations of pampas deer and to determine whether recent structuring has occurred in different locations. New studies should also analyze whether the structuring observed using mitochondrial markers for populations in Brazil (González *et al.*, 1998; Braga *et al.*, 2005), Argentina and Uruguay (González *et al.*, 1998) can be corroborated, since the exclusive use of mitochondrial markers may yield an artificial subdivision, an effect of female philopatry. Finally, it seems reasonable to affirm that the combination of techniques described here could easily be replicated in other species of Cervidae, with the appropriate methodological adjustments.

## Supplementary material

The following online material is available for this article:

Table S1 : List of the 50 samples used in the genetic characterization of pampas deer.
