# Supplementary material for: Genetic diversity of the pampas deer (Ozotoceros bezoarticus) population in the Brazilian Pantanal assessed by combining fresh fecal DNA analysis and a set of heterologous microsatellite loci
Source: Genet Mol Biol. 2017 Oct 2;40(4):774–80. doi: 10.1590/1678-4685-GMB-2016-0323 (PMC5738621; doi:10.1590/1678-4685-GMB-2016-0323)
Supplement: Supplementary file 1 [file 1415-4757-gmb-1678-4685-GMB-2016-0323-Suppl01.pdf]

Supplementary Material to "Genetic diversity of the Pampas deer (Ozotoceros bezoarticus) population from Brazilian Pantanal by combining fresh fecal DNA analysis and a set of heterologous microsatellite loci"

Table S1 - List of the 50 samples used in the genetic characterization of pampas deer, including category and genotype for six loci.

Sample	Category	Loci					
		BM757	RT09	RT01	Ca71	NVHRT03	NVHRT16
01	Adult female	178/194	102/104	220/230	317/317	122/124	170/184
02	Adult male	174/178	-	230/230	-	110/110	-
03	Adult male	188/194	108/110	232/232	-	112/112	168/170
04	Adult male	188/196	104/106	220/228	317/317	-	168/180
05	Adult male	194/196	106/108	228/230	317/317	108/116	168/176
06	Adult male	198/200	102/110	224/230	317/317	108/120	160/168
07	Adult female	182/186	102/108	220/220	-	110/122	164/182
08	Adult female	174/182	104/104	220/236	317/317	122/124	164/176
09	Adult male	184/188	102/110	222/232	317/317	112/114	166/184
10	Adult female	-	102/106	234/238	-	120/122	168/186
11	Adult male	196/198	104/108	220/234	317/317	122/122	154/182
12	Adult male	182/188	104/104	218/234	317/319	112/114	168/182
13	Adult male	188/196	102/104	220/220	-	110/112	168/170
14	Adult male	178/194	102/104	220/228	317/317	122/122	156/188
15	Adult male	172/184	102/104	220/232	-	108/108	166/170
16	Adult male	-	-	232/234	-	108/110	164/170
17	Adult male	188/194	102/108	230/230	317/317	-	168/178
18	Adult male	174/184	102/108	220/232	309/317	108/108	142/166
19	Adult female	182/184	-	220/230	317/321	108/108	-
20	Adult male	182/210	102/104	220/234	317/319	108/110	-
21	Adult female	182/194	104/104	220/236	317/317	122/124	178/190
22	Adult female	174/190	102/108	214/230	317/317	108/108	168/186
23	Adult female	182/210	102/104	220/220	-	110/112	184/184
24	Adult male	190/196	104/104	220/222	317/317	110/112	170/170
25	Adult male	-	102/102	220/230	317/317	-	-
26	Adult female	178/182	102/108	222/230	317/317	108/122	-
27	Adult male	184/196	102/108	220/222	317/317	112/126	170/176
28	Adult female	190/192	102/104	222/230	-	-	154/168
29	Adult female	178/188	-	222/236	317/317	120/126	176/178
30	Adult female	184/188	-	230/234	317/321	108/112	142/168
31	Adult male	188/196	102/102	220/236	317/319	108/108	166/178
32	Adult male	178/186	102/102	222/232	319/319	108/126	164/170
33	Adult female	186/194	108/108	214/220	-	108/126	168/182
34	Adult female	-	106/108	220/222	-	110/124	164/178
35	Adult male	176/180	102/104	226/230	-	114/114	-
36	Adult female	182/188	102/104	220/234	319/319	-	182/182
37	Adult male	182/202	102/104	214/238	317/317	120/122	156/178
38	Adult male	186/200	102/102	222/232	317/317	108/120	154/192
39	Adult male	178/186	102/102	226/236	319/319	-	-
40	Adult female	190/190	102/104	214/230	-	110/126	168/168
41	Adult male	188/188	104/104	222/230	317/317	110/114	154/180
42	Adult female	-	104/104	232/232	317/317	108/126	156/168
43	Adult female	178/198	102/104	220/226	-	110/124	-

Sample	Category	Loci					
		BM757	RT09	RT01	Ca71	NVHRT03	NVHRT16
44	Adult female	200/200	104/104	232/232	317/317	108/126	156/168
45	Adult male	-	102/104	218/220	-	-	166/168
46	Adult male	188/200	-	220/230	315/315	118/120	164/168
47	Adult female	182/190	-	232/232	317/317	108/134	182/184
48	Adult female	186/200	-	222/224	317/317	112/114	182/186
49	Adult female	178/178	102/104	232/238	317/317	112/122	178/182
50	Adult female	188/188	102/104	-	-	112/114	164/170
